# Clinical implications of squamous cell carcinoma in the colon and rectum: A comprehensive analysis from the National Cancer Database

**DOI:** 10.1111/codi.70074

**Published:** 2025-04-16

**Authors:** Metincan Erkaya, Cigdem Benlice, Kamil Erozkan, Emre Gorgun, Mehmet Ayhan Kuzu

**Affiliations:** ^1^ Department of Colorectal Surgery Digestive Disease and Surgery Institute, Cleveland Clinic Cleveland Ohio USA; ^2^ Department of General Surgery, School of Medicine Ankara University Ankara Turkey

**Keywords:** colon cancer, oncological outcomes, overall survival, rectal cancer, squamous cell carcinoma

## Abstract

**Aim:**

Squamous cell carcinoma (SCC) of the colon and rectum represents an exceptionally rare manifestation of gastrointestinal malignancy. The primary aim of this study was to elucidate baseline characteristics of colon SCC and its impact on overall survival (OS) in comparison with rectal SCC.

**Method:**

We conducted a retrospective analysis utilizing the National Cancer Database encompassing patients diagnosed with SCC of the colon and rectum from 2004 to 2019. Propensity score matching was employed to facilitate the comparison of OS between patients diagnosed with rectal and colon SCC who underwent surgery across four different tumour stages, with each stage evaluated individually.

**Results:**

A cohort comprising 249 colon SCC cases and 5398 rectal SCC cases was analysed. Colon SCC patients, compared with rectal SCC patients, were older (mean age 66 ± 13 vs. 62 ± 12 years, *p* < 0.05), predominantly male (44% vs. 30%, *p* < 0.05), more frequently diagnosed at an advanced stage (Stage IV: 51% vs. 16%, *p* < 0.05) and demonstrated a higher proportion of poorly differentiated to undifferentiated tumours (51% vs. 30%, *p* < 0.05). Treatment methods were also different, with 58% of colon SCC cases receiving surgical intervention compared with only 25% of rectal SCCs. In matched data, rectal SCC had a higher 5‐year OS rate across all stages except Stage 4: Stage I (colon 70%, rectum 79%), Stage II (colon 46%, rectum 49%) and Stage III (colon 44%, rectum 45%), Stage IV (colon 15%, rectum 12%).

**Conclusion:**

This study provides the largest cohort of patients with colon and rectal SCC to date and their distinct clinical and survival data acquired from a national database. The colon SCC group exhibited worse prognosis than rectal SCC patients.

**What does this paper add to the literature?**

This study advances colon squamous cell carcinoma (SCC) beyond the case report era, presenting the largest nationwide cohort analysis. By distinguishing colon from rectal SCC, it identifies distinct clinicopathological features, treatment patterns and worse outcomes in colon SCC, addressing a critical knowledge gap emphasizing the need for standardized management strategies.

## INTRODUCTION

Colorectal squamous cell carcinoma (SCC) is an exceptionally uncommon neoplasm, with an incidence ranging from 0.1 to 0.25 per 1000 diagnosed colorectal carcinomas [1, 2, 3]. Over the past decade, the management of anal SCC has undergone a paradigm shift with the introduction of the Nigro protocol [4]. However, a concrete consensus on managing colorectal SCC has yet to be reached. This is due to the rarity of SCC of the rectum, and its manifestation in the colon is even more unusual. Recent years have seen an increase in published literature on rectal SCC. An analysis of the Surveillance Epidemiology and End Results (SEER) database involving 999 cases of rectal SCC demonstrated an overall survival (OS) benefit with the use of chemoradiation therapy (CRT), while surgical resection of the primary tumour alone showed no such OS advantage [5]. Corroborating these findings, the current National Cancer Database (NCDB) study also found higher OS in Stage II and III rectal cancer in the chemoradiation‐only cohort compared with the surgery‐only cohort [6]. Given the scarcity of colon SCC cases, clinical data, therapeutic patterns and survival outcomes are mostly limited to individual case reports. To date, large population‐based analyses of colon SCC have not been reported.

Due to the uncommon nature of colorectal SCC, understanding the prognosis and optimal treatment for colorectal SCC is crucial. A comprehensive stage‐wise analysis, examining various treatment options such as surgery and CRT, and their sequential effects on survival, is undoubtedly needed to better understand the disease prognosis and survival outcomes. The aim of this study was to reveal demographic information, prognosis and the role of different treatment approaches for the management of colon and rectal SCC. We hypothesized that colon and rectal SCCs are distinct entities with potential differences in demographics, therapeutic patterns and survival outcomes.

## METHOD

### Patient selection and statistical analysis

A retrospective analysis was conducted utilizing data from the NCDB, encompassing patients diagnosed with primary colon and rectal SCC between 2004 and 2019. Patients with SCC, as defined by the histological codes 8070–8076, were included in this study. Patients who had histological confirmation through microscopic examination of tissue specimens were selected, ensuring the accuracy of the diagnosis based on tissue evidence. Patients with incomplete medical records or missing information about their chemotherapy or radiotherapy regimens were excluded from the study.

A total of 249 patients with primary SCC of the colon, from the caecum to the sigmoid colon, and 5398 patients with rectal SCC were identified. The demographics and pathological characteristics of colon and rectal SCC were compared between the two groups. Various therapeutic approaches, including surgery, chemotherapy and radiotherapy, were assessed individually across colon and rectal SCC to evaluate their impact on survival outcomes. Furthermore, we compared demographics and clinical presentations between colon and rectal SCC patients who underwent surgery to identify potential distinct characteristics between these groups.

To account for potential differences in demographics and clinical presentation, we performed propensity‐adjusted analyses and compared the therapeutic modalities and OS patterns of colon SCC with those of rectal SCC. For propensity score matching, a standardized difference greater than 0.10 was defined as a threshold for determining whether there is a meaningful imbalance between groups. Covariates included in the model were age at diagnosis, sex, Charlson–Deyo comorbidity score and year of diagnosis. After propensity score matching, the study found that all covariates were well balanced between the groups, indicating that the matching process was successful in achieving distributive balance. A 1:7, 1:2, 1:3, 1:1 matching was performed using the nearest‐neighbour method for pathological Stages I, II, III and IV, respectively, using the ‘MatchIt’ package in R. This varying matching ratio strategy was employed to maximize statistical power while maintaining a balanced comparison. This analysis enabled us to compare survival outcomes between colon and rectal SCC in matched patient groups who underwent surgery, using a stage‐wise approach and minimizing potential confounding factors.

Statistical analysis was conducted using descriptive statistics, and categorical variables among the groups were compared using the chi‐square test or Fisher's exact test, while Student's *t*‐test was employed for continuous variables. Kaplan–Meier survival analyses were conducted on both the unmatched and matched databases, comparing colon and rectal SCC. Three‐ and five‐year OS rates were analysed with different subsets regarding various therapeutic approaches. Statistical analysis was carried out using R version 4.2.3.

### Primary and secondary outcomes

The primary objective of this study was to determine the various therapeutic approaches employed in the management of colon and rectal SCC and to analyse survival outcomes based on the different therapeutic patterns. The secondary objective was to investigate whether colon and rectal SCC are distinct entities with potential differences in demographics, therapeutic patterns and survival outcomes. Subsequently, subgroup analyses were performed in the rectum and colon separately to explore potential discrepancies in OS rates.

### NCDB

The NCDB is a wide‐ranging clinical oncology database that collects data from hospital registries across the United States. It stands as one of the country's biggest cancer registries, incorporating almost 34 million cases from over 1500 facilities accredited by the Commission on Cancer. Its main objectives are surveillance and enhancing quality in cancer care, covering about 72% of all newly identified malignancies in the United States while also housing more than 34 million historical records [7]. Institutional review board or ethics committee approval, as well as written consent, were not necessary for this study due to its retrospective design and the use of publicly available NCDB data, which consists of de‐identified patient information.

## RESULTS

### Baseline characteristics

A cohort comprising 249 cases of colon SCC and 5398 cases of rectal SCC was analysed. Colon SCC patients, compared with rectal SCC patients, were older (mean age 66 ± 13 years vs. 62 ± 12 years, *p* < 0.05), less frequently Caucasian (83.9% vs. 86.8%, *p* < 0.05) and had more comorbidities as indicated by Charlson–Deyo scores 2 or more  (13.2% vs. 7.0%, *p* < 0.05). Both groups were predominantly female (56% vs. 70%, *p* < 0.05). Colon SCC patients were more frequently diagnosed at advanced stages (Stage IV, 51% vs. 16%, *p* < 0.05) and demonstrated a higher proportion of poorly differentiated to undifferentiated tumours (51% vs. 30%, *p* < 0.05) compared with rectal SCC patients. (Table 1).

**TABLE 1:** Demographics and pathological characteristics of squamous cell carcinoma.

Characteristic		Colon SCC (*n* = 249)	Rectal SCC (*n* = 5398)
Age (years)	Mean ± SD	66.9 ± 13.4	62.2 ± 12.3
	Median (IQR)	68 (21)	61 (16)
Sex, *n* (%)	Male	111 (44.6)	1644 (30.5)
	Female	138 (55.4)	3754 (69.5)
Race, *n* (%)	Caucasian	209 (83.9)	4673 (86.6)
	African‐American	32 (12.9)	546 (10.1)
	Other/unknown	8 (3.2)	179 (3.3)
Year of diagnosis, *n* (%)	2004–2007	46 (18.5)	874 (16.2)
	2008–2011	59 (23.7)	1214 (22.5)
	2012–2015	73 (29.3)	1582 (29.3)
	2016–2019	71 (28.5)	1728 (32.0)
Charlson–Deyo score, *n* (%)	0	168 (67.5)	4313 (79.9)
	1	48 (19.3)	706 (13.1)
	2	21 (8.4)	166 (3.1)
	3 or more	12 (4.8)	213 (3.9)
Grade, *n* (%)	Well differentiated	7 (2.8)	210 (3.9)
	Moderately differentiated	43 (17.3)	1592 (29.5)
	Poorly differentiated	121 (48.6)	1616 (29.9)
	Undifferentiated	8 (3.2)	52 (0.9)
	Unknown	70 (28.1)	1928 (35.8)
Stage, *n* (%)	Stage I	22 (8.8)	1585 (29.4)
	Stage II	48 (19.3)	1167 (21.6)
	Stage III	51 (20.5)	1746 (32.3)
	Stage IV	128 (51.4)	900 (16.7)
Chemotherapy, *n* (%)	Yes	88 (35.3)	4017 (74.4)
	Chemotherapy therapy before surgery	6	288
	Chemotherapy therapy after surgery	42	504
	Sequence unknown	40	3225
	No	155 (62.3)	1232 (22.8)
	Unknown	6 (2.4)	149 (2.8)
Radiotherapy, *n* (%)	Yes	32 (12.9)	3936 (72.9)
	Radiation therapy before surgery	6	316
	Radiation therapy after surgery	12	578
	Sequence unknown	14	3042
	No	211 (84.7)	1245 (23.1)
	Unknown	6 (2.4)	217 (4.0)
Surgery, *n* (%)	Surgery of the primary site was performed	145 (58.2)	1334 (24.7)
	Surgery of the primary site was not performed	104 (41.8)	4064 (75.3)
	It was not part of the planned first course treatment	91	3596
	It was contraindicated due to patient risk factors (comorbid conditions, advanced age, etc.)	8	111
	The patient died prior to planned or recommended surgery	1	22
	It was recommended by the patient's physician, but was not performed as part of the first course of therapy. No reason was noted in patient record	0	53
	It was recommended by the patient's physician, but this treatment was refused by the patient, the patient's family member or the patient's guardian. The refusal was noted in patient record	1	66
	Surgery of the primary site was recommended, but it is unknown if it was performed. Further follow‐up is recommended	1	89
	It is unknown whether surgery of the primary site was recommended or performed	2	127

Abbreviations: IQR, interquartile range; SCC, squamous cell carcinoma.

### Therapeutics and survival analysis

Treatment methods also differed, with 58% of colon SCC patients receiving surgical intervention compared with only 25% of rectal SCC patients. The primary reason for not performing surgery at the primary site was that it was not part of the planned first course of treatment for either colon or rectal SCC. Additionally, 35.3% of colon SCC patients received chemotherapy compared with 74.4% of rectal SCC patients, while 12.9% of colon SCC patients received radiation compared with 72.9% of rectal SCC patients (Table 1).

**FIGURE 1:**
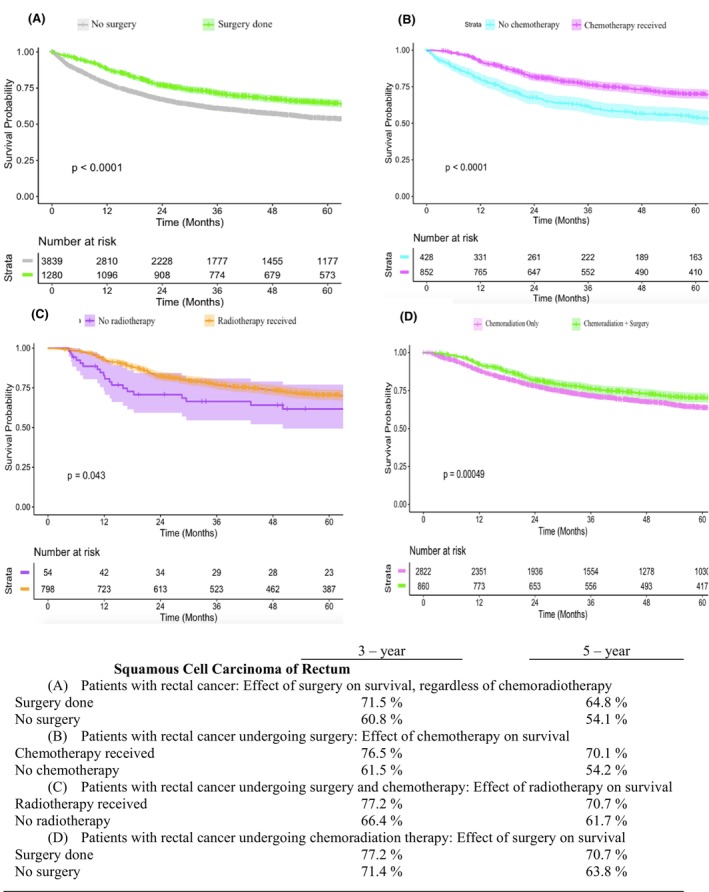
Kaplan–Meier estimates of overall survival for rectal squamous cell carcinoma patients across various treatment modalities.

**FIGURE 2:**
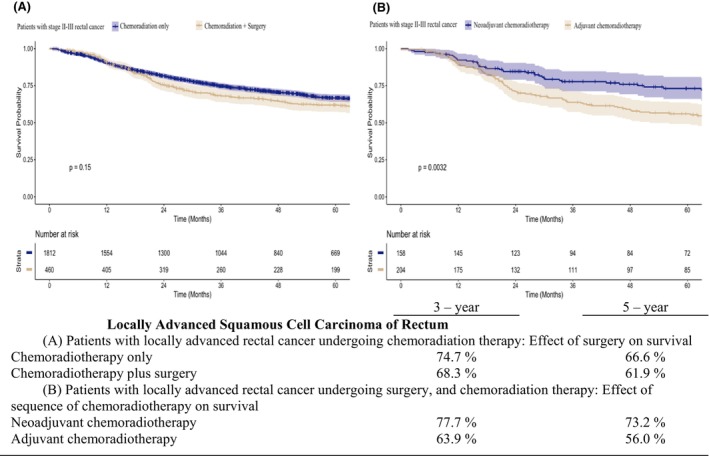
Kaplan–Meier estimates of overall survival for rectal squamous cell carcinoma patients in Stages II–III across various treatment modalities.

**FIGURE 3:**
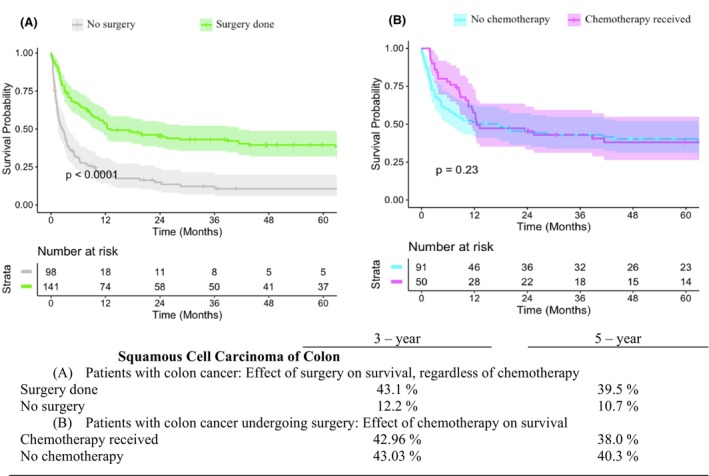
Kaplan–Meier estimates of overall survival for colon squamous cell carcinoma patients across various treatment modalities.

We conducted further analysis to compare OS rates for different therapeutic patterns in patients with rectal and colon SCC. For patients with rectal SCC in Stages I–IV, the 5‐year OS rate for those who underwent surgery was 64.8% compared with 54.1% for those who did not undergo surgery (*p* < 0.05) (Figure 1A). Among patients with rectal SCC who underwent surgery, CRT had a positive effect on survival (Figures 1B,C). For patients with rectal SCC who received CRT, the 5‐year OS rate was 70.7% for those who also underwent surgery, compared with 63.8% for those who did not (*p* < 0.05) (Figure 1D). However, subgroup analysis of rectal SCC patients showed that locally advanced rectal SCC patients who received chemoradiation only had slightly better OS than those receiving chemoradiation plus surgery, although this difference was not statistically significant (66.6% vs. 61.9%, *p* = 0.15) (Figure 2A). For patients with locally advanced rectal SCC who underwent both surgery and CRT, the sequence of treatments significantly affected the 5‐year OS rate, with neoadjuvant CRT resulting in better outcomes than adjuvant CRT (73.2% vs. 56.0%, *p* < 0.05). (Figure 2B) For patients with colon SCC in Stages I–IV, the 5‐year OS rate for those who underwent surgery was 39.5% compared to 10.7% for those who did not undergo surgery (*p* < 0.05) (Figure 3).

From the general cohort, we focused on patients who underwent surgical intervention. Among those who underwent surgery, 145 patients had colon SCC, with 141 having complete CRT information for analysis. For rectal SCC, 1334 patients underwent surgery, of whom 1280 had complete CRT data available. The stage distribution for colon SCC patients who underwent surgery (Stage I 15 patients, 10.6%; Stage II 42 patients, 29.8%; Stage III 44 patients, 31.2%; Stage IV 40 patients, 28.4%) is more advanced compared with rectal SCC patients who underwent surgery (Stage I 611 patients, 47.7%; Stage II 316 patients, 24.7%; Stage III 281 patients, 22.0%; Stage IV 72 patients, 5.6%) (Table 2).

**TABLE 2:** Demographic and pathological characteristics of patients with squamous cell carcinoma undergoing surgery, presented in a stage‐wise approach.

Characteristic	Matched cohort											
	Stage I			Stage II			Stage III			Stage IV		
	Colon (*n* = 15)	Rectum (*n* = 105)	SMD//*p*‐value	Colon (*n* = 42)	Rectum (*n* = 84)	SMD//*p*‐value	Colon (*n* = 44)	Rectum (*n* = 132)	SMD//*p*‐value	Colon (*n* = 36)	Rectum (*n* = 36)	SMD//*p*‐value
Age (years), mean ± SD	68 ± 8.6	64.5 ± 11.3	0.175	70.2 ± 14.4	67.2 ± 11.8	0.23	64.3 ± 15.2	63.5 ± 12.9	0.78	64.1 ± 13.7	61.5 ± 13.2	0.4
Age (years), median (IQR)	68 (12.5)	65 (17)		71 (19.25)	69 (17)		65.6 (25.75)	65 (19.5)		63 (18.25)	59.5 (20.75)	
Age (years), *n* (%)^a^												
<65	6 (40.0)	52 (49.5)	−0.19//0.68	14 (33.3)	27 (32.1)	0.03//1	20 (45.5)	65 (49.2)	−0.08//0.79	20 (55.6)	22 (61.1)	−0.11//0.81
≥65	9 (60.0)	53 (50.5)	0.19//0.68	28 (66.6)	57 (67.9)	0.03//1	24 (54.5)	67 (50.8)	0.08//0.79	16 (44.4)	14 (38.9)	0.11//0.81
Sex, *n* (%)^a^												
Male	9 (60.0)	67 (63.8)	−0.08//1	11 (26.2)	25 (29.8)	0.08//0.83	23 (52.3)	59 (44.7)	0.15//0.49	17 (47.2)	17 (47.2)	0.00//1
Female	6 (40.0)	38 (36.2)	0.08//1	31 (73.8)	59 (70.2)	−0.08//0.83	21 (47.7)	73 (55.3)	−0.15//0.49	19 (52.8)	19 (52.8)	0.00//1
Year of diagnosis, *n* (%)^a^												
2004–2007	4 (26.66)	30 (28.5)	−0.04//0.73	10 (23.8)	19 (22.6)	0.03//0.99	11 (25.0)	37 (28.0)	−0.07//0.95	8 (22.2)	7 (19.5)	0.07//0.99
2008–2011	4 (26.66)	28 (26.7)	0.00//0.73	8 (19.1)	15 (17.9)	0.03//0.99	10 (22.7)	31 (23.5)	−0.02//0.95	8 (22.2)	8 (22.2)	0.00//0.99
2012–2015	4 (26.66)	36 (34.3)	−0.17//0.73	14 (33.3)	30 (35.7)	−0.05//0.99	12 (27.3)	36 (27.3)	0.00//0.95	8 (22.2)	9 (25.0)	−0.07//0.99
2016–2019	3 (20.0)	11 (10.5)	0.24//0.73	10 (23.8)	20 (23.8)	0.00//0.99	11 (25.0)	28 (21.2)	0.088//0.95	12 (33.4)	1233 (33.3)	0.00//0.99
Race, *n* (%)												
Caucasian	14 (93.3)	89 (84.8)	0.6	34 (80.9)	76 (90.5)	0.25	36 (81.8)	117 (88.6)	0.5	28 (77.77)	29 (80.6)	0.26
African‐American	1 (6.7)	11 (10.5)		7 (16.7)	6 (7.1)		5 (11.4)	10 (7.6)		8 (22.22)	5 (13.9)	
Other/unknown	0 (0)	5 (4.7)		1 (2.4)	2 (2.4)		3 (6.8)	5 (3.8)		0 (0)	3 (5.5)	
Charlson–Deyo score, *n* (%)^a^												
0–1	13 (86.7)	92 (87.6)	−0.03//1	34 (81.0)	74 (88.1)	−0.18//0.42	38 (86.4)	120 (90.9)	−0.13//0.56	33 (91.66)	35 (97.22)	−0.21//0.6
2 or more	2 (13.3)	13 (12.4)	0.03//1	(19.0)	10 (11.9)	0.18//0.42	6 (15.6)	12 (9.1)	0.13//0.56	3 (8.33)	1 (2.77)	0.21//0.6
Grade, *n* (%)^a^												
Well to moderately differentiated	7 (46.66)	43 (41.0)	0.11//0.92	14 (33.3)	30 (35.7)	−0.05//0.76	12 (27.3)	44 (33.3)	−0.14//0.72	5 (13.9)	4 (11.1)	0.08//0.94
Poorly differentiated to undifferentiated	4 (26.66)	31 (29.5)	−0.06//0.92	24 (57.2)	49 (58.3)	−0.02//0.76	29 (65.9)	78 (59.1)	0.14//0.72	25 (69.4)	26 (72.2)	−0.06//0.94
Unknown	4 (26.66)	31 (29.5)	−0.06//0.92	4 (9.5)	5 (6.0)	0.12//0.76	3 (6.8)	10 (7.6)	−0.03//0.72	6 (16.7)	6 (16.7)	0.00//0.94
Radiotherapy, *n* (%)												
Yes	1 (6.6)	59 (56.2)	<0.001	9 (21.4)	56 (66.66)	<0.001	6 (13.6)	93 (70.5)	<0.001	2 (5.6)	16 (44.4)	<0.001
No	14 (93.4)	46 (43.8)		33 (78.6)	28 (33.33)		38 (86.4)	39 (29.5)		34 (94.4)	20 (55.6)	
Chemotherapy, *n* (%)												
Yes	2 (13.3)	55 (52.4)	0.01	10 (23.8)	60 (71.4)	<0.001	23 (52.3)	99 (75.0)	0.008	14 (38.9)	19 (52.8)	
No	13 (86.7)	50 (47.6)		32 (76.2)	24 (28.6)		21 (47.7)	33 (25.0)		22 (61.1)	17 (47.2)	

*Note*: SMD//*p*‐value: SMD represents the standardized mean difference (SMD) between groups before the //, and the *p*‐value represents the statistical significance of the difference after //. Covariates marked with superscript ‘a’ are represented by SMD//*p*‐value, while others simply show their corresponding *p*‐value.

^a^ Covariates included in the match model were age at diagnosis, sex, Charlson–Deyo comorbidity score, and year of diagnosis. Subset data were created individually for each stage.

**FIGURE 4:**
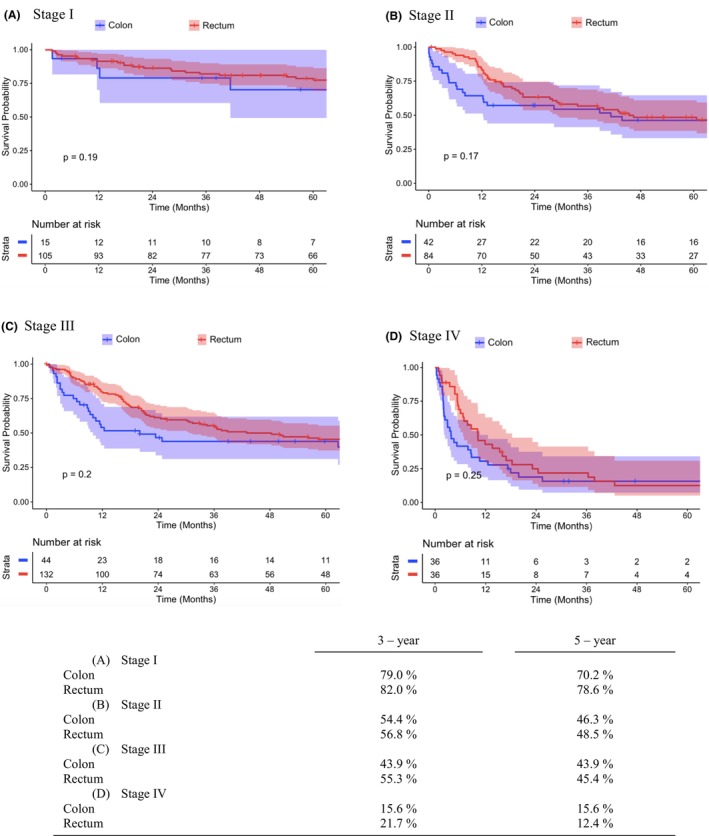
Kaplan–Meier estimates of overall survival for propensity‐matched cohorts of colon and rectal squamous cell carcinoma patients who underwent surgery across different stages.

In the unmatched cohort (Table S1), colon SCC was associated with worse OS rates across most stages compared with its rectal counterpart (Figure S1). Survival analysis showed that colon SCC had still worse survival rates than rectal SCC, even after adjusting for variables. In matched data, rectal SCC had higher 5‐year OS rates across all stages apart from Stage IV: Stage I (colon 70% vs. rectum 79%, *p* = 0.19), Stage II (colon 46% vs. rectum 49%, *p* = 0.17) and Stage III (colon 44% vs. rectum 45%, *p* = 0.20), Stage IV (colon 15% vs. rectum 12%, *p* = 0.25) (Figure 4). Further analysis of patients with R0 resection margins revealed a similar trend. For Stage I, 5‐year OS rates were 75.7% for colon SCC and 85.4% for rectal SCC (*p* = 0.074). In Stage II, rates were 49.5% for colon SCC and 59.4% for rectal SCC (*p* = 0.14). For Stage III, this trend reversed, with colon SCC showing slightly better outcomes: 50.8% for colon vs. 46.7% for rectum (*p* = 0.68). The stage‐wise anatomical distribution of colon SCC is presented in Table S2.

## DISCUSSION

This nationwide cohort study demonstrates that patients with rectal SCC have better outcomes than those with colon SCC, with significant differences observed in stage distribution and overall survival. While over half of the colon SCC patients underwent surgery, only a quarter of those with rectal SCC had surgical intervention. Most rectal SCC patients were treated with CRT, whereas the predominant treatment modality for colon SCC was surgery. Survival analysis revealed a significant advantage for patients with rectal SCC compared with those with colon SCC, even after adjusting for baseline characteristics between colon and rectal SCC, except for Stage IV disease. These findings highlight potential differences in disease processes between colon and rectal SCC, despite both being rare entities.

The demographic and clinical characteristics of colon and rectal SCC exhibited variances, with colon SCC patients being older, more frequently male and having a higher Charlson–Deyo score. Furthermore, individuals with colon SCC were commonly diagnosed at advanced stages and had a higher occurrence of poorly differentiated to undifferentiated tumours compared with those with rectal SCC. It is noteworthy that in Stage IV colon SCC, approximately 67% of patients did not undergo surgical intervention. This observation warrants further investigation into the factors influencing treatment decisions and outcomes in advanced stages of this rare malignancy. Given the high proportion of patients with inoperable disease at diagnosis, our findings demonstrate the critical need for new diagnostic strategies to facilitate early detection of colon SCC, potentially improving treatment options and patient outcomes.

SCC of the colorectal region presents a spectrum of disease with varying prognoses and treatment approaches. Anal SCC, the most common site for SCC in the colorectal area, has seen significant advancements in management with the introduction and widespread adoption of the Nigro protocol [4, 8]. In contrast, rectal and colon SCC, being rarer entities, lack such well‐established protocols. Comparative studies have shown that anal SCC generally presents at earlier stages and has a better prognosis than rectal SCC. However, this survival advantage diminishes in advanced stages, with similar outcomes observed for Stage III disease between anal and rectal SCC. Furthermore, when patients receive a combination of surgery and CRT, the survival gap between anal and rectal SCC narrows, suggesting that rectal SCCs may be less radiosensitive and more reliant on multimodal treatment approaches [9, 10]. Colon SCC represents the rarest and most aggressive form within this spectrum. Compared with rectal SCC, colon SCC is typically diagnosed at a more advanced stage and carries a poorer prognosis. The lack of standardized treatment protocols for colon SCC may contribute to its relatively poor outcomes compared with its rectal and anal counterparts.

The treatment of colorectal SCC has primarily relied on isolated case series from individual institutions [11]. However, recent years have witnessed significant advances, particularly in studies focusing on rectal SCC. Various modalities, including surgery alone, surgery followed by CRT, definitive CRT and CRT followed by surgery, have been described for colorectal SCC based on these studies. The role of CRT as primary therapy or in conjunction with surgery is more prominent in the rectum, while surgery is more commonly employed in the colon.

In the context of rectal SCC, our analysis indicates that surgical intervention is an effective treatment modality. Among patients with rectal SCC who underwent surgery, patients receiving CRT demonstrated a superior OS rate compared with those without CRT. For locally advanced rectal SCC patients undergoing surgery and CRT, neoadjuvant CRT conferred a survival advantage over adjuvant CRT. In this group of patients with locally advanced rectal SCC it was noteworthy that those who received only CRT showed no significant difference in OS compared with those who underwent both CRT and surgery. Numerous studies have supported the use of CRT as the main treatment approach, reserving surgical intervention for instances of local failure or recurrence as a salvage therapy [12, 13, 14]. Kommalapati et al. [6] found a higher OS in Stage II and III disease in the CRT‐only cohort compared with the surgery‐only cohort. However, no such difference in OS was noted in Stage I disease, whether managed with CRT alone or definitive surgical therapy alone. Additionally, Goffredo et al. [15] analysed the SEER database and concluded that radiation treatment should be preferred over surgery for rectal SCC. Notably, Chiu et al. [5] found no survival benefit of surgery on OS, recommending nonsurgical radiotherapy‐based treatments instead. In contrast, Steinemann et al. [16] reported that patients with regional rectal SCC who underwent multimodal therapy, including surgery, experienced improved OS and longer disease‐free intervals than those treated without surgery. However, this survival benefit was not observed in patients with localized or distant disease.

Given the rarity of colon SCC, clinical data, treatment approaches and survival outcomes are predominantly derived from isolated case reports [17, 18, 19, 20]. Consequently, the management of this entity relies heavily on small case series from individual institutions, describing various treatment modalities. These include surgery alone, surgery followed by adjuvant chemotherapy, definitive CRT and chemotherapy followed by surgical resection. Some case reports employing various combination therapy regimens, including adjuvant chemotherapy following surgery, have successfully demonstrated the utility of chemotherapy for colon SCC [10, 21]. Our study demonstrates that surgical intervention significantly improves survival rates for patients with colon SCC across Stages I–IV. The addition of chemotherapy did not have a significant impact on survival rates for colon cancer patients undergoing surgical resection when compared with those who did not receive chemotherapy.

The retrospective nature of our study and its reliance on NCDB analysis poses certain limitations. We were unable to investigate the impact of different treatment protocols on progression‐free survival and disease recurrence. Using a comprehensive database with a significant sample size is important for analysing therapeutic trends, identifying prognostic factors and conducting overall survival analyses for this rare malignancy. Our study included patients who underwent surgery, utilizing the NCDB Participant User File for colon and rectal cancer. We did not include rectosigmoid junction SCC due to its distinct nature, imprecise localization and its presence in a different NCDB Participant User File. Moreover, the NCDB does not provide detailed information on chemotherapy regimens, which restricts our ability to analyse the impact of specific chemotherapy approaches on survival outcomes. In the context of rectal SCC, due to the limitations of this dataset, we were unable to apply Williams' criteria [22] to distinguish rectal from anal SCCs. Consequently, precise differentiation between these two entities was not possible. Additionally, our study was limited by the inability of the NCDB to identify upper, middle and lower rectal SCC, as this may also influence the way the SCC behaves. Furthermore, the relationship between anal and rectal SCC and human papillomavirus and HIV infection was not assessed.

## CONCLUSION

This study presents the largest cohort of colon and rectal SCC patients to date, offering distinct clinical and survival data derived from the NCDB. Colon SCC predominantly affects an older demographic, with a higher proportion of male patients and individuals with preexisting comorbidities compared with rectal SCC. This rare malignancy frequently presents at advanced stages, characterized by poorly differentiated or undifferentiated tumours. While over half of colon SCC patients underwent surgery, only a quarter of rectal SCC patients received surgery. Notably, the colon SCC group exhibited worse survival rates than the rectal SCC patients. Given the lack of concrete consensus regarding the treatment of this SCC disease entity, there is a clear need for larger databases and prospective studies beyond case‐based analysis.

### Future directions

Future research in colorectal SCC should focus on several key areas. Prospective, multicentre studies are crucial to establish standardized treatment protocols for both colon and rectal SCC, moving beyond the current reliance on case reports and small series. Developing new diagnostic strategies for earlier detection of colon SCC is essential for improving treatment options and patient outcomes.

## AUTHOR CONTRIBUTIONS

**Metincan Erkaya:** Conceptualization; writing – original draft; writing – review and editing; project administration; formal analysis; methodology; validation; data curation. **Cigdem Benlice:** Supervision; writing – review and editing; writing – original draft; conceptualization; methodology; validation. **Kamil Erozkan:** Writing – review and editing; conceptualization; methodology; visualization. **Emre Gorgun:** Writing – original draft; conceptualization; methodology; supervision; writing – review and editing. **Mehmet Ayhan Kuzu:** Writing – review and editing; validation; methodology; supervision; project administration; conceptualization; investigation.

## FUNDING INFORMATION

This is not a sponsor‐funded study.

## CONFLICT OF INTEREST STATEMENT

Dr Emre Gorgun is a consultant for Boston Scientific, DiLumen, Olympus and Vascular Technology. Other authors do not have any conflicts of interest or financial ties to disclose for this work. This is not a sponsor‐funded study.

## Supporting information


Figure S1.

Table S1.

Table S2.

## Data Availability

The NCDB is a joint project of the Commission on Cancer of the American College of Surgeons and the American Cancer Society. The data used in the study are derived from a deidentified NCDB file. The American College of Surgeons and the Commission on Cancer have not verified and are not responsible for the analytic or statistical methodology employed or the conclusions drawn from these data by the investigators.
