# Supplementary material for: Clinical implications of squamous cell carcinoma in the colon and rectum: A comprehensive analysis from the National Cancer Database
Source: Colorectal Dis. 2025 Apr 16;27(4):e70074. doi: 10.1111/codi.70074 (PMC12003965; doi:10.1111/codi.70074)
Supplement: Supplementary file 1 — Figure S1. [file CODI-27-0-s003.docx]

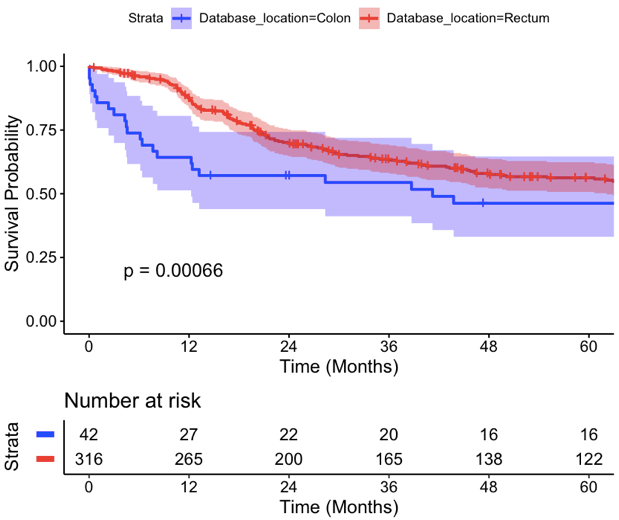

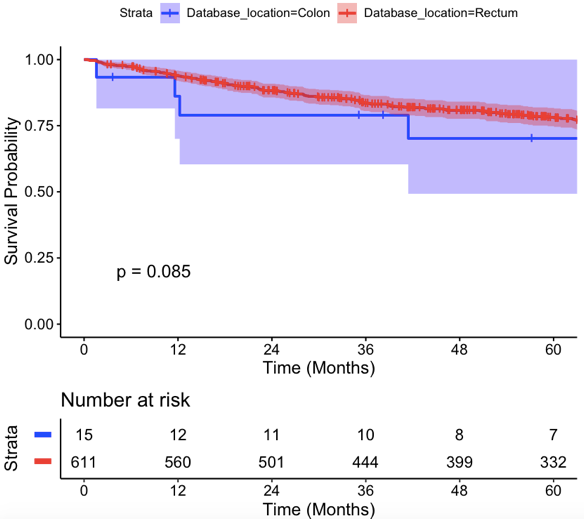


(B)

(A)


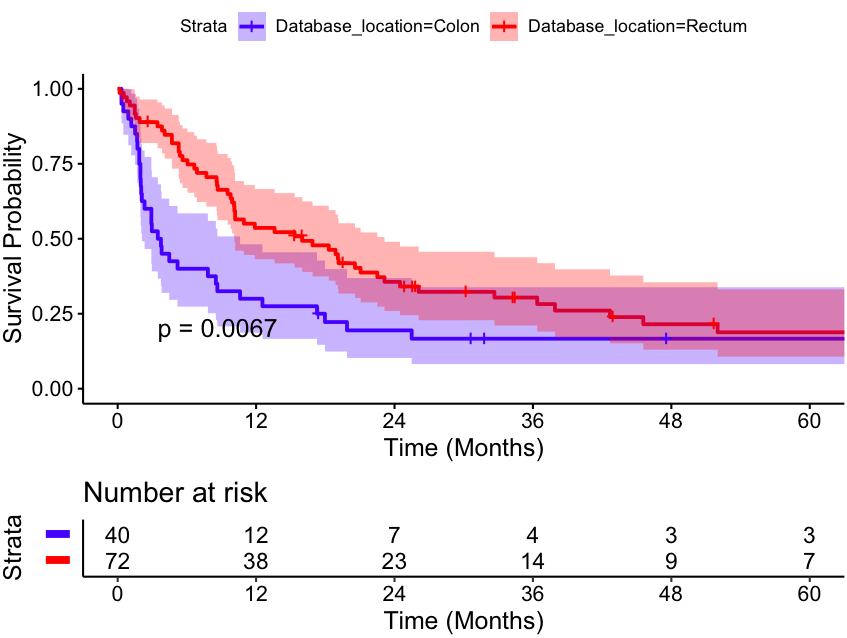

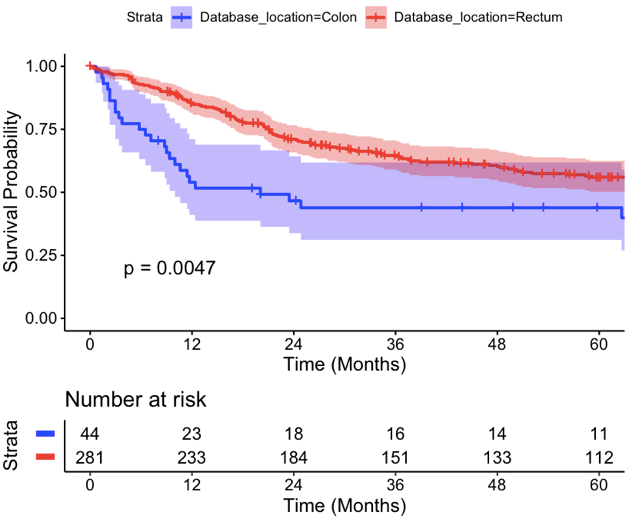


(C)

(D)

|  | 3 – year |  | 5 – year |
| --- | --- | --- | --- |
| 1. Stage I |  |  |  |
| Colon | 79,0 % |  | 70,2 % |
| Rectum | 83,5 % |  | 78,2 % |
| 1. Stage II |  |  |  |
| Colon | 54,4 % |  | 46,3 % |
| Rectum | 63,5 % |  | 56,2 % |
| 1. Stage III |  |  |  |
| Colon | 43,9 % |  | 43,9 % |
| Rectum | 64,6 % |  | 56,0 % |
| 1. Stage IV |  |  |  |
| Colon | 16,7 % |  | 16,7 % |
| Rectum | 30,4 % |  | 18,8 % |
| **Supplemental Figure 1.** Kaplan -Meier Estimates of Overall Survival (OS) for Unmatched Cohort of Colon and Rectal Squamous Cell Carcinoma (SCC) Across Different Stages | | | |
