# Supplementary material for: Clinical implications of squamous cell carcinoma in the colon and rectum: A comprehensive analysis from the National Cancer Database
Source: Colorectal Dis. 2025 Apr 16;27(4):e70074. doi: 10.1111/codi.70074 (PMC12003965; doi:10.1111/codi.70074)
Supplement: Supplementary file 2 — Table S1. [file CODI-27-0-s001.docx]

| **Supplemental Table 1** Demographic and Pathological Characteristics of Patients with Squamous Cell Carcinoma Undergoing Surgery | | | | | | | | | | | | | | | | |
| --- | --- | --- | --- | --- | --- | --- | --- | --- | --- | --- | --- | --- | --- | --- | --- | --- |
| Characteristics |  | **Unmatched Cohort** | | | | | | | | | | | | | | |
|  |  | Stage I | | |  | Stage II | | |  | Stage III | | |  | Stage IV | | |
|  |  |  |  |  |  |  |  |  |  |  |  |  |  |  |  |  |
|  |  |  |  |  |  |  |  |  |  |  |  |  |  |  |  |  |
|  |  | Colon  N=15 | Rectum  N=611 | SMD //  p value |  | Colon  N=42 | Rectum  N=316 | SMD //  p value |  | Colon  N=44 | Rectum  N=281 | SMD //  p value |  | Colon  N=40 | Rectum  N=72 | SMD //  p value |
| Age,  mean ± SD |  | 68  ± 8.6 | 62.8  ± 12.9 | 0.037 |  | 70.2  ± 14.4 | 60.4  ± 12.1 | <0.001 |  | 64.3  ± 15.2 | 61.3  ± 12.3 | 0.22 |  | 64.2  ± 13.3 | 60.4  ± 11.6 | 0.13 |
| Age,  median (IQR) |  | 68  (12.5) | 62  (17) |  |  | 71  (19.3) | 60  (18) |  |  | 65.5  (25.8) | 61  (17) |  |  | 63  (18.3) | 59  (15.3) |  |
| Age, n (%)* |  |  |  |  |  |  |  |  |  |  |  |  |  |  |  |  |
| < 65 |  | 6  (40.0) | 358  (58.6) | -0.38 // 0.24 |  | 14  (33.3) | 205  (64.9) | -0.67 //  <0.001 |  | 20  (45.5) | 177  (63.0) | -0.35 //  0.04 |  | 23  (57.5) | 50  (69.4) | -0.24 //  0.29 |
| >= 65 |  | 9  (60.0) | 253  (41.4) | 0.38 // 0.24 |  | 28  (66.6) | 111  (35.1) | 0.67 //  <0.001 |  | 24  (54.5) | 104  (37.0) | 0.35 //  0.55 |  | 17  (42.5) | 22  (30.6) | 0.24 //  0.29 |
| Sex, n (%)* |  |  |  |  |  |  |  |  |  |  |  |  |  |  |  |  |
| Male |  | 9  (40.0) | 185  (30.3) | 0.61 //  0.03 |  | 11  (26.2) | 101  (32.0) | 0.13 //  0.56 |  | 23  (52.3) | 78  (27.8) | 0.49 //  0.002 |  | 19  (47.5) | 26  (36.1) | -0.23 //  0.33 |
| Female |  | 6  (60.0) | 426  (69.7) | -0.61 //  0.03 |  | 31 (73.8) | 215  (68.0) | -0.13 //  0.56 |  | 21  (47.7) | 203  (72.2) | -0.49 //  0.002 |  | 21  (52.5) | 46  (63.9) | 0.23 //  0.33 |
| Year of diagnosis, n (%)* | | |  |  |  |  |  |  |  |  |  |  |  |  |  |  |
| 2004-2007 |  | 4  (26.66) | 152  (24.9) | 0.04 //  0.99 |  | 10  (23.8) | 81  (25.6) | -0.04 //  0.59 |  | 11  (25.0) | 57  (20.3) | 0.11 //  0.88 |  | 8  (20.0) | 18  (25.0) | -0.13 //  0.33 |
| 2008-2011 |  | 4  (26.66) | 160  (26.2) | 0.01 //  0.99 |  | 8  (19.1) | 87  (27.5) | -0.21 //  0.59 |  | 10  (22.7) | 64  (22.8) | 0.00 //  0.88 |  | 12  (30.0) | 11  (15.3) | 0.32 //  0.33 |
| 2012-2015 |  | 4  (26.66) | 157  (25.7) | 0.02 //  0.99 |  | 14  (33.3) | 83  (26.3) | 0.15 //  0.59 |  | 12  (27.3) | 90  (32.0) | -0.11 //  0.88 |  | 8  (20.0) | 16  (22.2) | -0.06 //  0.33 |
| 2016-2019 |  | 3  (20.0) | 142  (23.2) | -0.08 //  0.99 |  | 10  (23.8) | 65  (20.6) | 0.08 //  0.59 |  | 11  (25.0) | 70  (24.9) | 0.002 //  0.88 |  | 12  (30.0) | 27  (37.5) | -0.16 //  0.33 |
| Race, n (%) |  |  |  |  |  |  |  |  |  |  |  |  |  |  |  |  |
| Caucasian |  | 14  (93.3) | 524  (85.8) | 0.65 |  | 34  (80.9) | 274  (86.7) | 0.57 |  | 36  (81.8) | 250  (89.0) | 0.34 |  | 31  (77.5) | 60  (83.3) | 0.1 |
| African-American |  | 1  (6.7) | 63  (10.3) |  |  | 7  (16.7) | 35  (11.1) |  |  | 5  (11.4) | 22  (7.8) |  |  | 9  (22.5) | 8  (11.1) |  |
| Other/ Unknown |  | 0  (0) | 24  (3.9) |  |  | 1  (2.4) | 7  (2.2) |  |  | 3  (6.8) | 9  (3.2) |  |  | 0  (0) | 4  (5.6) |  |
| Charlson-Deyo Score, n (%)* | | |  |  |  |  |  |  |  |  |  |  |  |  |  |  |
| 0-1 |  | 13  (86.7) | 584  (95.6) | -0.26 //  0.32 |  | 34  (81.0) | 296  (93.7) | -0.32 //  0.009 |  | 38  (86.4) | 266  (94.7) | -0.24 //  0.079 |  | 37  (92.5) | 67  (93.1) | -0.02 //  1 |
| 2 or more |  | 2  (13.3) | 27  (4.4) | 0.26 //  0.32 |  | (19.0) | 20  (6.3) | 0.32 //  0.009 |  | 6  (15.6) | 15  (5.3) | 0.24 //  0.079 |  | 3  (7.5) | 5  (6.9) | 0.02 //  1 |
| Grade, n (%)* |  |  |  |  |  |  |  |  |  |  |  |  |  |  |  |  |
| Well to moderately differentiated |  | 7  (46.66) | 291  (47.6) | -0.02 //  0.92 |  | 14  (33.3) | 140  (44.3) | -0.23 //  0.015 |  | 12  (27.3) | 98  (34.9) | -0.17 //  <0.001 |  | 5  (12.5) | 22  (30.5) | -0.55 //  0.007 |
| Poorly differentiated to undifferentiated |  | 4  (26.66) | 183  (30.0) | -0.07 //  0.92 |  | 24  (57.2) | 110  (34.8) | 0.45 //  0.015 |  | 29  (65.9) | 106  (37.7) | 0.59 //  <0.001 |  | 29  (72.5) | 30  (41.7) | 0.69 //  0.007 |
| Unknown |  | 4  (26.66) | 137  (22.4) | 0.1 //  0.92 |  | 4  (9.5) | 66  (20.9) | -0.39 //  <.015 |  | 3  (6.8) | 77  (27.4) | -0.82 //  <0.001 |  | 6  (15.0) | 20  (27.8) | -0.36 //  0.007 |
| Radiotherapy, n (%) | | |  |  |  |  |  |  |  |  |  |  |  |  |  |  |
| Yes |  | 1  (6.6) | 359  (58.8) | <0.001 |  | 9  (21.4) | 236  (74.7) | <0.001 |  | 6  (13.6) | 224  (79.7) | <0.001 |  | 2  (5.0) | 41  (56.9) | <0.001 |
| No |  | 14  (93.4) | 252  (41.2) |  |  | 33  (78.6) | 80  (25.3) |  |  | 38  (86.4) | 57  (20.3) |  |  | 38  (95.0) | 31  (43.1) |  |
| Chemotherapy, n (%) | | |  |  |  |  |  |  |  |  |  |  |  |  |  |  |
| Yes |  | 2  (13.3) | 342  (56.0) | 0.003 |  | 10  (23.8) | 242  (76.6) | <0.001 |  | 23  (52.3) | 277  (80.8) | 0.76 |  | 15  (37.5) | 41  (56.9) | 0.076 |
| No |  | 13  (86.7) | 269  (44.0) |  |  | 32  (76.2) | 74  (23.4) |  |  | 21  (47.7) | 54  (19.2) |  |  | 25  (62.5) | 31  (43.1) |  |
| SMD // p value: SMD represents the standardized mean difference (SMD) between groups before the //, and the p-value represents the statistical significance of the difference after //. Covariates marked with asterisk are represented SMD // p value:, while others simply show their corresponding p-values.  *Covariates included in the model were age at diagnosis, sex, Charlson comorbidity score, and year of diagnosis. Subset data were created individually for each stage. | | | | | | | | | | | | | | | | |
