# Supplementary material for: Clinical implications of squamous cell carcinoma in the colon and rectum: A comprehensive analysis from the National Cancer Database
Source: Colorectal Dis. 2025 Apr 16;27(4):e70074. doi: 10.1111/codi.70074 (PMC12003965; doi:10.1111/codi.70074)
Supplement: Supplementary file 3 — Table S2. [file CODI-27-0-s002.docx]

| **Supplemental Table 2** Stage-wise distribution of tumor locations in colon squamous cell carcinoma | | | | | | | | |  |  |
| --- | --- | --- | --- | --- | --- | --- | --- | --- | --- | --- |
| **Primary Location, n (%)** |  | Stage I |  | Stage II |  | Stage III |  | Stage IV |  | Combined Stage |
| Cecum |  | 6 |  | 6 |  | 10 |  | 17 |  | **39** |
| Ascending colon |  | 3 |  | 8 |  | 11 |  | 24 |  | **46** |
| Hepatic flexure |  | 2 |  | 2 |  | 2 |  | 9 |  | **15** |
| Transverse colon |  | 1 |  | 2 |  | 4 |  | 9 |  | **16** |
| Splenic flexure |  | 0 |  | 3 |  | 1 |  | 4 |  | **8** |
| Descending colon |  | 0 |  | 1 |  | 2 |  | 3 |  | **6** |
| Sigmoid colon |  | 8 |  | 19 |  | 15 |  | 38 |  | **80** |
| Colon, NOS |  | 2 |  | 5 |  | 2 |  | 22 |  | **31** |
| Overlapping lesion of colon |  | 0 |  | 2 |  | 4 |  | 2 |  | **8** |
| **Location sites, n (%)** |  |  |  |  |  |  |  |  |  |  |
| Left-sided colon cancer |  | 8 |  | 23 |  | 18 |  | 45 |  | **94 (37.8)** |
| Right-sided colon cancer |  | 12 |  | 18 |  | 27 |  | 59 |  | **116 (46.6)** |
| Others |  | 2 |  | 7 |  | 6 |  | 24 |  | **39 (15.6)** |
| **Total** |  | **22** |  | **48** |  | **51** |  | **128** |  | **249** |
| Right-sided of the colon includes the cecum, ascending colon, hepatic flexure, and transverse colon.  Left-sided colon cancer can occur in the splenic flexure, descending colon, and sigmoid colon.  Other location sites include colon, NOS (not otherwise specified), and overlapping lesions of the colon. | | | | | | | | | | |
